# Strategies for Deliberate Induction of Immune Tolerance in Liver Transplantation: From Preclinical Models to Clinical Application

**DOI:** 10.3389/fimmu.2020.01615

**Published:** 2020-07-31

**Authors:** Naoki Tanimine, Masahiro Ohira, Hiroyuki Tahara, Kentaro Ide, Yuka Tanaka, Takashi Onoe, Hideki Ohdan

**Affiliations:** ^1^Department of Gastroenterological and Transplant Surgery, Graduate School of Biomedical and Health Sciences, Hiroshima University, Hiroshima, Japan; ^2^Medical Center for Translational and Clinical Research Hiroshima University Hospital, Hiroshima, Japan; ^3^Kure Medical Center and Chugoku Cancer Center, National Hospital Organization, Kure, Japan

**Keywords:** tolerance, liver, transplantation, immunosuppression, immunomonitoring

## Abstract

The liver exhibits intrinsic immune regulatory properties that maintain tolerance to endogenous and exogenous antigens, and provide protection against pathogens. Such an immune privilege contributes to susceptibility to spontaneous acceptance despite major histocompatibility complex mismatch when transplanted in animal models. Furthermore, the presence of a liver allograft can suppress the rejection of other solid tissue/organ grafts from the same donor. Despite this immune privilege of the livers, to control the undesired alloimmune responses in humans, most liver transplant recipients require long-term treatment with immune-suppressive drugs that predispose to cardiometabolic side effects and renal insufficiency. Understanding the mechanism of liver transplant tolerance and crosstalk between a variety of hepatic immune cells, such as dendritic cells, Kupffer cells, liver sinusoidas endothelial cells, hepatic stellate cells and so on, and alloreactive T cells would lead to the development of strategies for deliberate induction of more specific immune tolerance in a clinical setting. In this review article, we focus on results derived from basic studies that have attempted to elucidate the immune modulatory mechanisms of liver constituent cells and clinical trials that induced immune tolerance after liver transplantation by utilizing the immune-privilege potential of the liver.

## Introduction

Liver transplantation is currently a highly successful treatment for end-stage liver disease. It is well-known that liver allografts are tolerogenic, and stable grafts can be maintained across major histocompatibility complex (MHC) barriers without immunosuppression (IS) in some species (1–3). Furthermore, the presence of a liver allograft can suppress the rejection of other solid tissue grafts (e.g., heart and skin) from the same donor; hence, the liver favors introduction of immune tolerance rather than immunity (2, 4). Such a capacity of the transplanted liver to establish tolerance in an allogeneic host has been ascribed to the unique features and anatomical structure of hepatic constituent cells. In a clinical setting, however, the majority of liver transplant (LT) recipients require long-term immunosuppressive drug treatment to control the alloimmune responses. The undesired adverse effects of life-long IS remain a concern, that is, an increased risk of chronic kidney disease, metabolic disorders, infection and malignancy in LT recipients.

Safely minimizing or discontinuing IS without compromising liver allograft can be an attractive strategy to improve long-term survival after liver transplantation. For this purpose, significant efforts have been made to identify sensitive and specific biomarkers of immune tolerance in LT recipients or to establish reliable immune monitoring methods. Understanding the mechanism of the inherently tolerogenic nature of the liver would lead to the development of strategies for deliberate induction of more specific immune tolerance in clinical liver transplantation. Immune regulation in the liver is mainly controlled by a variety of antigen presenting cells (APCs), which spatiotemporally react with alloreactive T cells in LT recipients. In addition to professional APCs, such as dendritic cells (DCs), unique populations of non-professional APCs consisting of Kupffer cells, liver sinusoidal endothelial cells (LSECs), and hepatic stellate cells (HSCs) that express low levels of MHC class I/II and co-stimulatory molecules are resident in a steady-state liver. These cells are likely involved in fine-tuning the modulation of local and systemic tolerance and/or immunity after liver transplantation. In this review article, we focus on studies that attempted to elucidate the immune modulatory mechanisms of these APCs, and clinical trials that induced immune tolerance after liver transplantation by enjoying the immune-privilege potential of the liver.

## Role of Apcs in Immune Tolerance in Liver Transplantation

### Dendritic Cells

In mice, liver, but not other organ allografts, are accepted permanently and with donor specificity between many strain combinations, without the requirement for IS (3). It has been demonstrated that donor-derived DC precursors of liver allografts can be propagated in granulocyte macrophage colony-stimulating factor (GMC-SF) from the bone marrow (BM) or spleen of unmodified LT recipients in mouse model, suggesting that bidirectional leukocyte migration and donor cell chimerism contribute to liver graft acceptance and acquired transplantation tolerance (5). A recent study supported the assumption that DCs contribute to tolerance by demonstrating that recipients of DC-depleted liver allograft showed acute rejection while those receiving non-manipulated liver allograft showed indefinite acceptance in a transgenic mouse model (6). It has been previously shown that Flt3 ligand administration, which increases interstitial DCs and their interleukin (IL)-12 production, abrogated the acceptance of transplanted liver, and IL-12 neutralization markedly prolonged graft survival in mice receiving the Flt3 ligand (7, 8). In addition, it has been reported that the transmembrane adaptor protein, DNAX-activating protein of 12 kDa (DAP12), negatively regulates liver myeloid DC maturation and stimulation ability, and *DAP12*^−/−^ livers are rejected in relation to increased pro-inflammatory cytokines including IL-12p40 (9). These results suggest that DAP12 expression by liver DCs may be critical for the induction of tolerance. Hence, donor-derived DCs assuredly contribute to tolerance status; however, it likely depends on the DC subset and inflammatory status after transplantation. Recently, it has been reported that DCs contribute to tolerance in another mechanism in context of regulatory T cells (Tregs) IS, i.e., antigen (Ag)-specific Tregs that are formed strong interactions with DCs, result in the removal of the Ag and MHC class II complex from DC surface and reducing DC's Ag-presenting capacity (10). This might be one mechanism of tolerance induction by DCs. Based on such knowledge obtained in the preclinical_models, clinical trial for operational tolerance using regulatory DCs has been conducted (11). As a result, it has been shown that infusion of donor-derived, *ex-vivo* generated regulatory DCs can achieve operational tolerance in patients after liver transplantation, encouraging tolerance induction strategy with regulatory DCs in the future.

### Liver Sinusoidal Endothelial Cells

**Figure 1:**
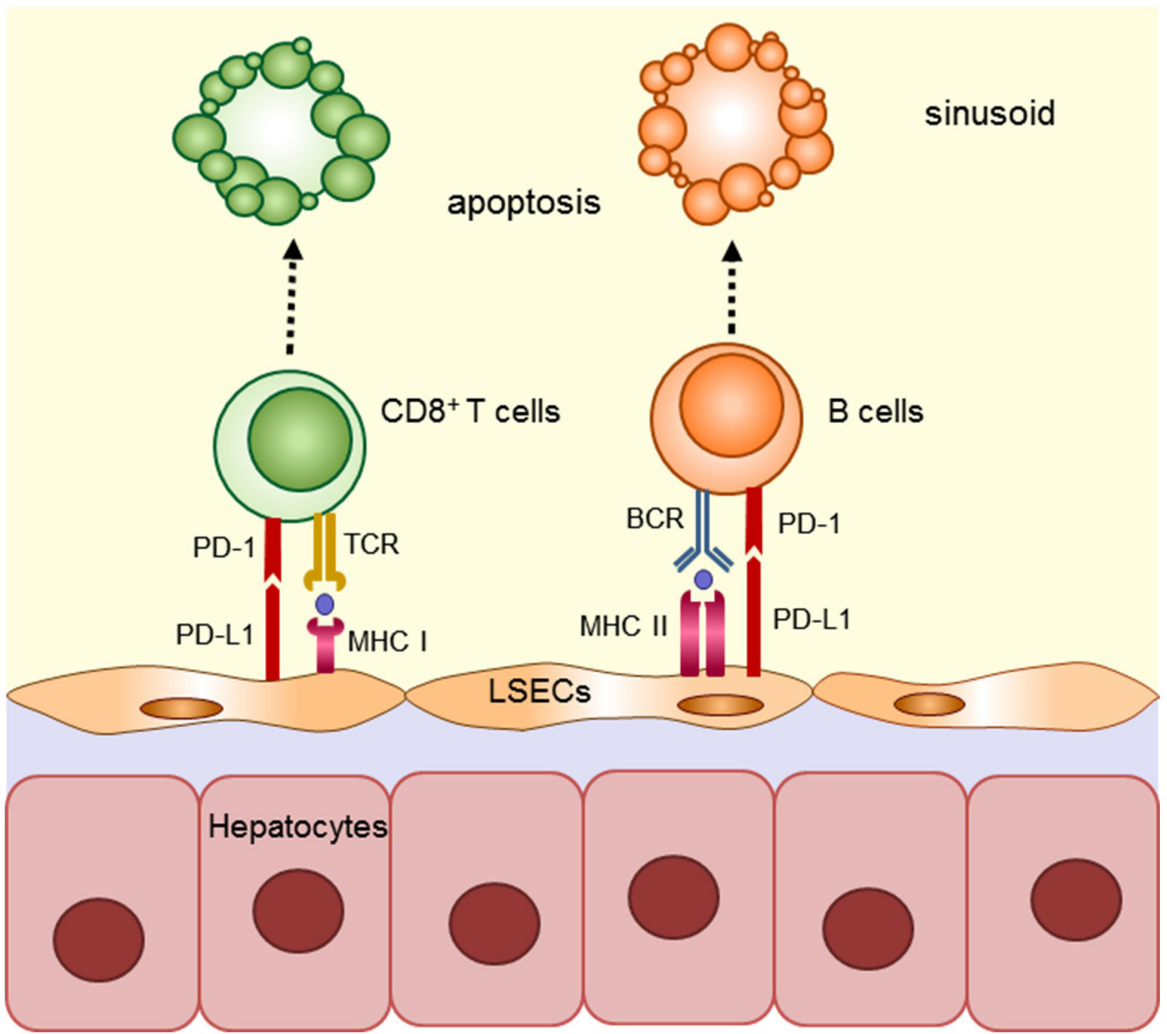
Mechanism implicated in regulating anti-donor immune cells by LSECs in grafted liver. LSECs constitutively express classes I and II and have the capacity for Ag presentation. LSECs contribute to the establishment of immunological tolerance in grafted liver by promoting apoptosis of donor-MHC reactive T and B cells through Ag-presentation and PD-1/PD-L1 signaling.

The sinusoids correspond to the capillaries of the liver, and have a more complex structure than ordinary capillaries. The diameter of the sinusoids is 5–7 μm, which is narrow enough to allow circulating lymphocytes to contact LSECs closely with effective immune interaction. In fact, LSECs constitutively express the molecules necessary for Ag presentation (CD80, CD86, CD40, and MHC classes I and II), and have the capacity for Ag presentation, which is not observed in endothelial cells of other organs (12). Furthermore, LSECs express Fas-ligand and programmed death-ligand (PD-L) 1, which has been recently attracted due to Nobel-prize winning checkpoint inhibitor studies (13–15). These molecules on LSECs induce apoptosis of reactive T cells, and suppress allo-reactive and Ag-specific T cells in a mouse model (12, 16–18). LSECs can also endocytose foreign Ag and suppress cognate T cells in allogeneic, exogenous, and cancer Ag models (16, 19, 20). The immunological suppressive capacity of LSECs was reported in an *in vitro* model (12, 21) as well as *in vivo* models (22). In these studies, chimeric livers, produced by adoptive transfer of allogeneic LSECs, induced suppression of allo-specific T cells *in vivo*; however, the suppressive effect of LSECs was attenuated by anti-PD-L1 antibody (Ab) during engraftment of allogeneic LSECs. Another study using a similar model, proved that LSECs have the ability to induce tolerance of carbohydrate reactive B cells through the PD-L1 pathway by demonstrating that chimeric α1,3-galactosyltransferase gene knockout (GalT) mice in which Gal-deficient LSECs were replaced with wild-type LSECs by adoptive transfer, lost the ability to produce anti-Gal Abs even after repeated immunization (23). This result suggests that LSECs also contribute to establishment of spontaneous tolerization of B cells in ABO-blood type incompatible liver transplantation. In a mouse orthotopic liver transplantation model, it has been reported that PD-L1 mediates the immune regulatory function of graft non-hematopoietic non-parenchymal cells including LSECs (24, 25). In this model, liver allografts from chimeric mice with *PD-L1*^+/+^ hematopoietic cells and *PD-L1*^−/−^ non-hematopoietic cells were rejected, whereas those from wild-type mice with *PD-L1*^+/+^ hematopoietic cells and P*D-L1*^+/+^ non-hematopoietic cells were accepted *in vivo*, suggesting that *PD-L1*^+/+^ non-hematopoietic cells, such as LSECs or stellate cells, likely contribute to the tolerogenicity of the liver via the PD-L1/PD-1 axis. In summary, these results suggest that LSECs contribute to the establishment of immunological tolerance in grafted liver by promoting apoptosis of donor-MHC reactive T and B cells through Ag-presentation and PD-1/PD-L1 signaling (Figure 1).

Notably, both in a mouse model and clinical living related liver transplantation, we have recently reported that portal hypertension enhances alloimmune responses, likely due to the impaired immune-suppression capacity of LSECs (26). In these studies, we demonstrated that expression of molecules necessary for Ag presentation and PD-L1, and the suppressive capacities of LSECs were decreased in portal hypertension. These results also strongly imply that LSECs contribute to the establishment of tolerance status after liver transplantation and importance of control of portal hypertension for achieving tolerance in liver transplantation.

### Heptic Stellate Cells (HSCs)

HSCs are pericytes found in the Disse space, which is a space between the sinusoid s and hepatocytes. HSCs are classified as fibroblasts, and are well-described for their important role for hepatic fibrosis and storage of vitamin A. It has been recently shown that HSCs also function as APCs (27). HSCs express CD1d, MHC class II, and CD86, which are integral for APC, and present Ag to reactive T cells. It has been reported that mouse and human HSCs express PD-L1, and activated HSCs markedly upregulate PD-L1 expression and induce T cell-hyporesponsiveness *in vitro* (28, 29). This immune-suppressive effect of HSCs is triggered by IFN-γ and regulates the MEK/ERK pathway (30). Furthermore, it has been recently reported that HSCs preferentially induce Foxp3^+^ Tregs by the production of retinoic acid (31). In an *in vivo* model, co-transplantation of HSCs effectively protects islet allograft from rejection through PD-L1 signaling (32). These results suggest that HSCs have immune suppressive features similar to LSECs and play an important role in tolerogenic status in the liver. Of note, HSCs may be related to pericytes or mesenchymal stem/stromal cells *in vivo* due to their genetic proximity and similarities of phenotype and differentiation potency (33–35). These cells have been shown to elicit very elaborate immunoregulatory effects (36–38). In fact, a phase I-II clinical study of infusion of MSC after deceased liver transplantation to achieve operational tolerance has been reported (39). This study also might encourage a clinical application of HSC.

## Other Basic Mechanisms of Immune Tolerance in Liver Transplantation Involving Breg Cells and Nkt Cells

### Regulatory B Cells

Recent studies have shown the existence of a distinct subset of B cells with immunomodulatory properties, which have been termed regulatory B cells (Bregs), analogous to Tregs. Bregs have been found to play a pivotal role in regulating immune responses involved in inflammation, autoimmunity, and malignancy (40). Their main mechanism of action is by promoting the development of Tregs while suppressing effector CD4^+^ and CD8^+^ T cells, primarily by secreting IL-10, IL-35, and transforming growth factor β (TGFβ), which produce donor-specific antibodies and induce antibody-mediated rejection. However, recent studies have indicated that Bregs, which possess antibody-independent effector functions, have the capacity to control or regulate immune responses to a transplanted organ (41, 42).

As one part of Breg cells, B cells were found to express PD-L1 and PD-L2, which are well-known to have a pivotal role in regulating autologous T cell-immune response in self-immunity by engaging PD-1, providing immune homeostasis and mediating the mechanisms of tolerance (43, 44). We have recently demonstrated that the unique B-1 cell subset expressing PD-L1 and PD-L2 inhibits alloimmune T cell responses in mice (45).

Although the role of Breg cells in immune tolerance in clinical liver transplantation remains to be elucidated, one study revealed that sirolimus could amplify Bregs and Tregs among LT recipients, which might be beneficial in mitigating the immune response (46). The role of Breg cells in liver transplantation is becoming increasingly understood, and tolerization relevant to Breg cells might be expected to be applied clinically.

### Natural Killer T Cells

Invariant natural killer T cells (iNKT cells), which express an invariant T cell receptor (TCR) α-chain and recognize lipids present on CD1d, secrete diverse cytokines (such as interferon-γ, IL-4, IL-5, IL-10, and IL-13) and influence many types of immune responses (47). In general, iNKT cells are non-circulating, tissue-resident lymphocytes, but the prevalence of different iNKT cell subsets differs markedly between tissues, that is, the liver, lungs, adipose tissue, and intestine (48). Among these tissues, iNKT cells are most frequent in the liver in both mice and humans.

In organ/tissue transplantation, iNKT cells play a significant immune-regulatory role in the maintenance of transplant tolerance to allografts (49–51). It has been demonstrated that CD40L/CD28 blockade fails to maintain tolerance to allograft in iNKT cell-deficient recipients mice, while peripheral transplant tolerance can be induced in wild-type recipients by that treatment (51). Consistently, it has been shown that liver allografts lacking iNKT cells manifested infiltration, hemorrhage and necrosis with significant reduction of graft survival and much less induction of tolerance compared with wild-type liver allograft in mice (52). Hence, iNKT cells, particularly donor-liver resident iNKT cells, are found to be immune regulatory cells that play a vital role in inducing spontaneous tolerance after allogeneic liver transplantation. In addition, we have also demonstrated that iNKT cells play a significant role in the immunosuppressive effects induced by LSECs on T cells with indirect allospecificity (53).

## Immunosuppression Withdrawal Trials

In 1993, Reyes et al. in the Pittsburgh group reported the first series of operational tolerant recipients whose allograft did not show functional deterioration after cessation of immunosuppressants (ISs) due to their mandatory requirements such as severe infection and malignancy (54). Operational tolerance is separately understood from immunological tolerance that is observed as no proof of immunological activity in the experimental model. After the Pittsburgh report, a total of 17 groups have reported their experience and trials (11 adult/4 pediatric/2 mix population) to pursue the ideal goal, transplant tolerance, which may allow the return of natural immunity and free them from the side effects of IS (Table 1) (54–75).

**Table 1:** Studies for spontaneous tolerance in liver transplantation.

**Institution**	**Published year**	**Living/Cadaver**	**Pediatric/Adult**	**Study design**	***n***	**Patient with S.E**.	**Baseline biopsy**	**Time since LT (criteria) yr**	**IS regimen**	**Success rate**	**Acute rejection (Chronic rejection)**	**Graft loss**	**Remarks**
Pittsburgh	1993 (54)	–	–	Case series reports	6	Yes	No	NA	NA	NA	NA	NA	First series report from Pittsburgh
	1995 (55) 1997 (56)	NA	Mix	Prospective	59 95	No	Yes	Mean 8.4 (>5)	14% Aza, 12% Tac 74% CsA	18/95 (19%)	25.4% (NR)	0	Two of PBC developed recurrence
King's College	1998 (57)	Cadaver	Adults	Prospective	18	Yes	No	Median 7 (–)	CsA and Aza	5/18 (27.7%)	28% (5.6%)	1/18 (5.6%)	Fewer HLA mismatch was associated with successful withdrawal. Previous rejection history and autoimmune original disease are risk factor
Kyoto	2001 (58)	Living	Pediatric	Partially prospective	26 (63)	Partially yes	No	NA (>2)	Tac	24/63 (38.1%)	12% (NR)	0	Biopsy at 4 year after weaning showed that 2 of 11 tolerant recipients had substantial bile duct atrophy and recovered by tacrolimus reinduction
	2002 (59)	Living	Mix	Prospective + retrospective	115	Partially yes	No	NA (>2)	Tac	16/67 (23.9%)	Non-protocol 25% Protocol 11.9%	0	None of clinical characteristics was identified as predictor of successful weaning
Marcia	2003 (60)	Cadaver	Adult	Prospective	9	No	Yes	Median 5.1 (>2)	CyA	3/9 (33%)	22% (NR)	0	Endothelial cell chimerism seems to have nothing to do with the induction of clinical tolerance in liver transplant patients
Stanford	2004 (61)	NA	Pediatric	Retrospective	38	Yes	No	NA	Steroid+CNI (Tac92%, CyA 8%)	8/38 (20.5%)	55.3% (5.3%)	2/38 (5.3%)	Two patients were retransplanted for chronic rejection
New Orleans	2005 (62)	Cadaver	Adult	Prospective	18	No	No	(>0.5)	Tac	1/18 (5.6%)	61% (NR)	0	Early induction of operational tolerance seems to be difficult
Miami	2005 (63)	Cadaver	Adult	RCT (donor BM)	105	No	No	Mean 4 (>3)	85% Tac 14% CsA	0%	67% (1.9%)	1/105 (0.95%)	Donor bone marrow infusion did not help successful completion of withdrawal
Rome	2006 (64) 2008 (65) 2013 (66)	Cadaver	Adult	Prospective	34	No	Yes	Mean 5.3 (>1)	CsA monotherapy	8/34 (23.4%)	76.4% (NR)	0	All HCV related recipients
Israel	2007 (67)	NA	Adult	RCT	26	No	No	Mean 4.3 vs. 5.0 (>2)	CsA +/–Aza, (Plednisone)	2/26 (7.7%)	UDCA+ 43% UDCA– 75%	0	3/4 AIH recipients had recurrence
Korea	2009 (68)	Mix	Pediatric	Retrospective	5	Yes	No	Median 3.8	NA	–	NR	0	Long term stable graft function and no rejection >1 yr were favorable findings for successful withdrawal
UCSF	2012 (69)	Living	Pediatric	Multi-center prospective	20	No	Yes	Mean 7.7 (>3)	CNI monotherapy	12/20 (60%)	36.8% (NR)	0	Later initiation of IS withdrawal after transplantation and less portal inflammation and total C4d score on screening biopsy were associated with successful withdrawal
Pamplona	2013 (70)	Cadaver	Adult	Prospective	24	Yes	Yes	Median 9.3 (>3)	NA	15/24(62.5%)	4.1% (41%)	0	Tolerant patients had a longer median interval between transplantation and inclusion in the study (156 vs. 71 months)
Barcelona	2013 (71)	Cadaver	Adult	Multi-center prospective	102	No	Yes	Median 8.6 (>3)	CNI mTOR inhibitor CSB	41/102 (40.2%)	56% (NR)	0	Time since transplantation, recipient age, and male gender were independent factor for successful withdrawal
	2014 (72)	Cadaver	Adult	Multi-center prospective	32	No	Yes	Median 7.2 (>3)	CNI +/–MMF or CBS	17/34 (50%)	44.1% (NR)	0	Persistent viral infections exert immunoregulatory effects that could contribute to the restraining of alloimmune responses
Taipei	2015 (73)	Mix	Pediatric	Single center retrospective	16	No	Yes	(>1 for Tx <1, > 2 for Tx > 1)	Tac monotherapy	5/15 (33%)	46.7% (NR)	0	Early recruitment was favorable factor predicting operational tolerance
Chicago	2019 (74)	Cadaver	Adult	Prospective	15	No	Yes	Mean 6.7 (>3)	Silorimus	8/15 (53.3%)	40% (NR)	0	mTOR inhibitor withdrawal had similarly succeeded in comparison with CNI withdrawal
Pennsylvania	2019 (75)	Cadaver	Adult	Multi-center RCT	77	No	Yes	Median 18 (>3)	Tac (91) CsA (2), MMF(2)	10/77 (13%)	40.3% (NR)	0	Withdrawal showed likely less eventful than maintenance group

*S.E., side effect; Aza, azathioprine; Tac, tacrolimus; CsA, cyclosporine A; NR, not reported; NA, not assessed; CNI, calcineurin inhibitor; RCT, randomized control study; CSB, costimulatory blockade; MMF, mycophenolate mofetil*.

Two early trials at Pittsburgh by Dr. Starzl and King's college by Dr. Williams and their colleagues revealed that attempting complete IS withdrawal could be successful in some recipients (19 and 27.7%, respectively), and long-surviving LT recipients were generally over-immunosuppressed (55–57). Since several experimental models have shown that donor chimerism can induce transplant tolerance (76–78), these trials and a randomized control trial (RCT) in Miami by Tryphonopoulos et al. (63) have assessed micro- and macro-chimerism as mechanisms of operational tolerance. However, donor-chimerism was not proven to be a mechanism of clinical operational tolerance. Later, Eason et al. at the New Orleans tried early induction of operational tolerance and showed that it seemed difficult to succeed, but still feasible with regard to reversal rejection events and subsequent graft survival (62). A similar finding has been shown in a recent multicenter trial with strict selection criteria and withdrawal protocol (75). As another risk factor for failure of complete IS withdrawal, recent episodes of rejection, autoimmune-related original disease were reported in early studies, and these factors are recognized as standard exclusion criteria for recent IS withdrawal trials (57, 67). Operational tolerance in pediatric recipients presented by Dr. Feng and her colleagues in San Francisco seems to show a relatively higher success rate compared to adult cases. This may be because of their immature immune system, but one of the other reasons could be more living donor cases, particularly parents who share the haplotype of HLA. Actually, data from living donor-related recipients are limited in adults. It may be a good candidate for investigating the mechanism of operational tolerance.

Currently, two IS withdrawal trails supported by the Immune Tolerance Network (ITN) leaded by Dr. Nepom are in operation. Recent trials achieving relatively high success rates of withdrawal by using strict selection criteria (69–72) showed that time after transplantation and age of recipients are the most impactful and common clinical factors of operational tolerance. These studies also suggested that exhausted T cells against hepatitis C virus (HCV) in HCV-related recipients and hyporesponsive T cells against polyclonal stimulation prior to withdrawal could contribute to the establishment of tolerance (70, 72). Based on these findings, multicenter IS withdrawal trial is currently being conducted by Dr. Markmann and his colleagues in Boston (NCT02533180, OPTIMAL) for evaluating donor-specific immune senescence and exhaustion as biomarker of operational tolerance in adults. Dr. Sanchez-Fueyo and his colleagues in Spain is conducting another trial (NCT02498977, LIFT) with a similar structure, but focused on exploring biomarkers in transcriptional signatures to identify operational tolerant recipients. The results of both trials could open a new gate to understand the mechanism of operational tolerance.

## Impact of DSA on Immunosuppression Withdrawal

The deleterious effect of donor-specific antibody (DSA) on LT recipients is increasingly recognized, but has not been well-defined. The DSA may cause two types of antibody-mediated rejection (AMR): one is acute AMR resulting in immunologically adverse consequence because of preformed DSA usually accompanied by cellular rejection in the early postoperative period, and the other is chronic AMR causing progressive fibrosis in the late phase after liver transplantation. A retrospective cohort study has shown that *de novo* DSA (dnDSA) is associated with rejection, graft loss, and patient death after liver transplantation, and one of the risk factors for developing dnDSA is inadequate IS (79). However, a recent IS withdrawal trial in adult primary LT patients (A-WISH trial, NCT00135694) has shown that there was no difference in the prevalence of dnDSA (especially HLA class II dnDSA) between IS maintenance and IS minimization (44.4% vs. 51.7%, respectively), and the prevalence was the highest after IS withdrawal was completed (66.7%) (75, 80). Interesting findings in prevalence have been reported that the majority (78.7%) of dnDSA was developed against HLA-DQ Ags, which included DQB1 (57.4%) and DQA1 (21.3%) chains independent of IS status, and dnDSA against HLA class I Ags increased only when patients were free of IS. From the view of pathogenicity, dnDSA detected in patients who failed IS withdrawal may be highly pathogenic compared to that in patients under IS maintenance and IS-free according to the prevalence of acute rejection rate (71.4, 25.0, and 16.7%, respectively) (80).

It is well-recognized that different IgG subclasses have unique characteristics, such as complement fixation potential or cellular binding capacity through Fc receptors (FcRs), which may affect their pathologic potential. IgG3 is known as the strongest complement activating capacity, followed by IgG1 and IgG2, while IgG4 is the only subclass that fail to fix complement. IgG3 and IgG1 bind to all three classes of FcRs (FcRI, FcRII, and FcRIII), while IgG4 binds to FcRII and FcRIII, and IgG2 binds only FcRII (81). These binding abilities have the potential to trigger functions such as antibody-dependent cell cytotoxicity, cytokine production, intracellular signaling, and initiation of cell recruitment and degranulation with various immune mediators (macrophages, NK cells, neutrophils, and B cells) (82). Jackson et al. recently examined whether DSA IgG subclass characteristics could identify subjects whose liver allografts exhibit subclinical graft injury with samples from a multicenter IS withdrawal study for pediatric LT recipients (iWITH, NCT01638559) (83). They reported that the HLA-class II IgG4 DSA profile was associated with a higher HLA mismatch, a subclinical histopathological phenotype characterized by interface activity, and a tissue transcriptional profile of rejection. Substantial IgG subclass analysis for DSA in a prospective study is expected for better understanding and management of the dynamic evolution of DSA maturation, mechanisms of injury, and entry points for intervention (84). DSA is produced against HLA mismatches and HLA Ags has been reported to have structural epitope that dominate the strength and specificity of binding antibody (85). Recently, it has been reported that HLA class II epitope mismatch, which was analyzed by HLA Matchmaker or the predicted indirectly recognizable human leukocyte antigen epitopes algorithm (PIRCHE-II), is correlated with a high risk of dnDSA formation after liver transplantation (86, 87). By using more detail data on HLA class II epitope mismatch related to donor recipients, the eligibility criteria for patient selection in early IS minimization or IS withdrawal trials may be sophisticated (12).

## Immune Monitoring to Personalize Immunosuppression Toward Tolerance

**Figure 2:**
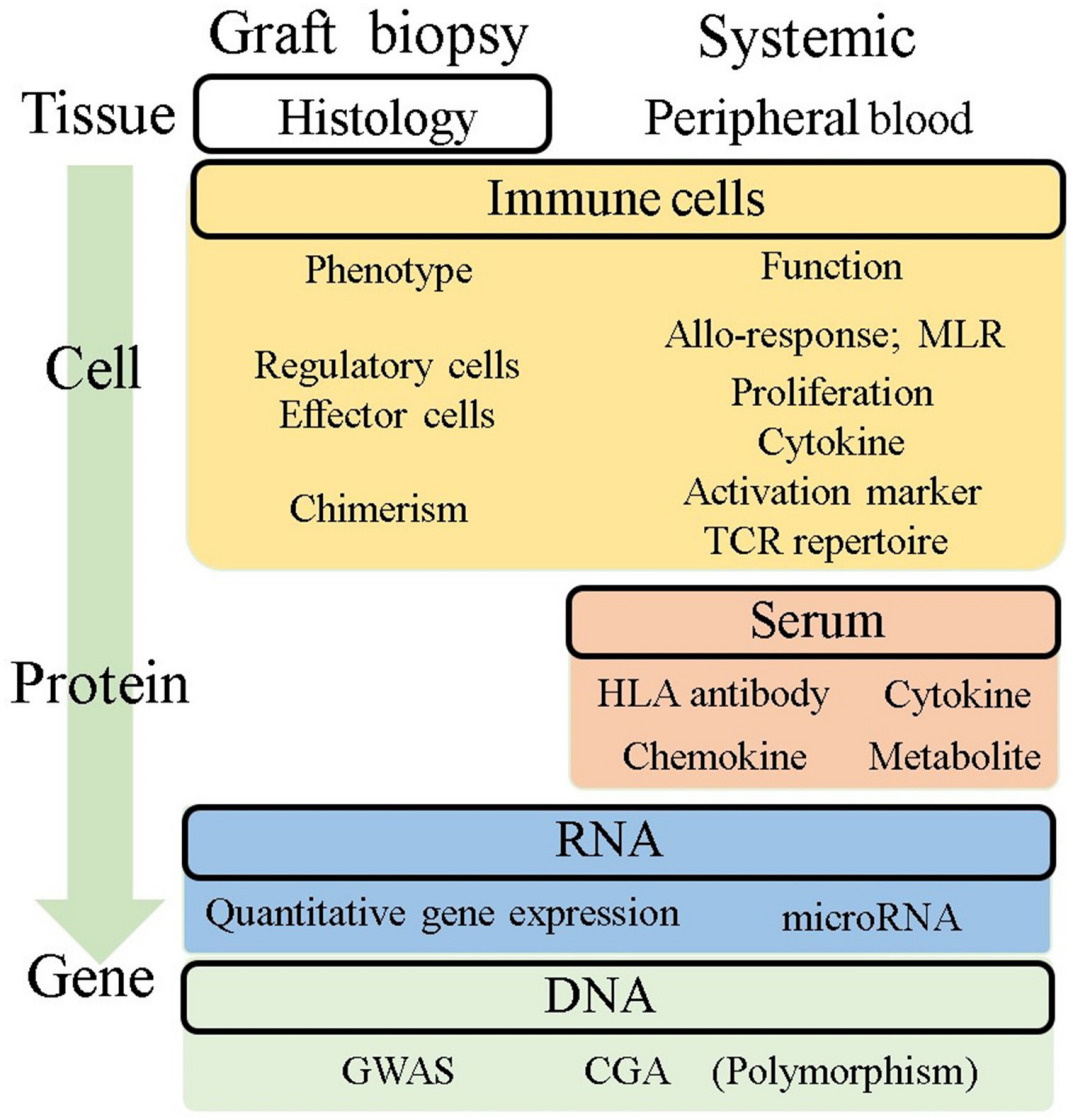
Potential immune monitoring application for tolerance in liver transplantation. This figure summarizes the readouts being investigated for their potential use for immune monitoring to understand what is happening in the allograft and predict tolerance. Histological assessment is a direct readout of allografts, but it is not enough to predict tolerance. Systemic information from peripheral blood has been investigated as an alternative because of its less invasive availability. The readouts were categorized into four groups based on their level of information, tissue, cell, protein, and gene. MLR, mixed lymphocyte reaction; GWAS, genome-wide association study; CGA, candidate gene approach.

Liver transplant recipients receive immunosuppressive therapy according to empirical protocols. Immune monitoring comprises candidate biomarkers capable of reflecting the donor-specific and non-specific net-activating state of the immune system, and can be dissected into tissue, cell, protein, and gene profiles with graft or systemic samples (Figure 2). Here, we summarize the potential tool for immune monitoring to personalize immunosuppressive therapy potentially toward operational tolerance.

As clinical information, histological findings regarding inflammation and fibrosis are the gold standard for the diagnosis of rejection. Intensive molecular analyses of biopsy specimens have shown that immune regulatory markers, such as IL-10, PD1, PDL1, BATF, TGFβ, and Foxp3 were significantly higher in tolerant patients (72). Intra-graft iron metabolism has also been identified in tolerant samples (88). Immunofluorescence staining revealed transient accumulation of CD4^+^FOXP3^+^ cells in tolerant recipients, along with the upregulation of immune regulatory genes (89). Although biopsy-based assessments are a valuable source of information on immunological status, it can be harmful because of their potential risk of complications. As candidates of safer biomarkers for successful withdrawal, the immune phenotype of peripheral blood has also been diligently investigated. Pittsburgh group reported that the increase in the ratio of plasmacytoid DCs to monocytoid DCs in peripheral blood was associated with successful withdrawal (90). Consistent with this finding, a study with a mammalian target of rapamycin (mTOR) inhibitor showed a higher proportion of tolerogenic DCs in tolerant recipients (74). An increasing number of regulatory T cell subsets (Tregs and gamma-delta T cells) and NK cells in peripheral blood are associated with tolerant recipients, which is consistent with their gene signatures (58, 65, 74, 91). A recent report has shown the kinetics of increasing Tregs/Th17 cell ratio over the clinical course as a predictor of the development of tolerance (92). These have the potential to be monitoring tools for tolerance, but further investigations are needed to validate their capacities. Pioneering studies for transplant tolerance has been conducted by “The One Study” consortium leading by Dr. Geissler and his colleagues. This consortium conducted harmonized cell therapy studies by multi-center to induce tolerance with standard immunosuppressive regimen and immune monitoring protocol, which allow to analyze different trial data under same platform. These approach also would be great helpful to build solid and universal foundation in clinical tolerance, which is observed to a limited extent.

**Figure 3:**
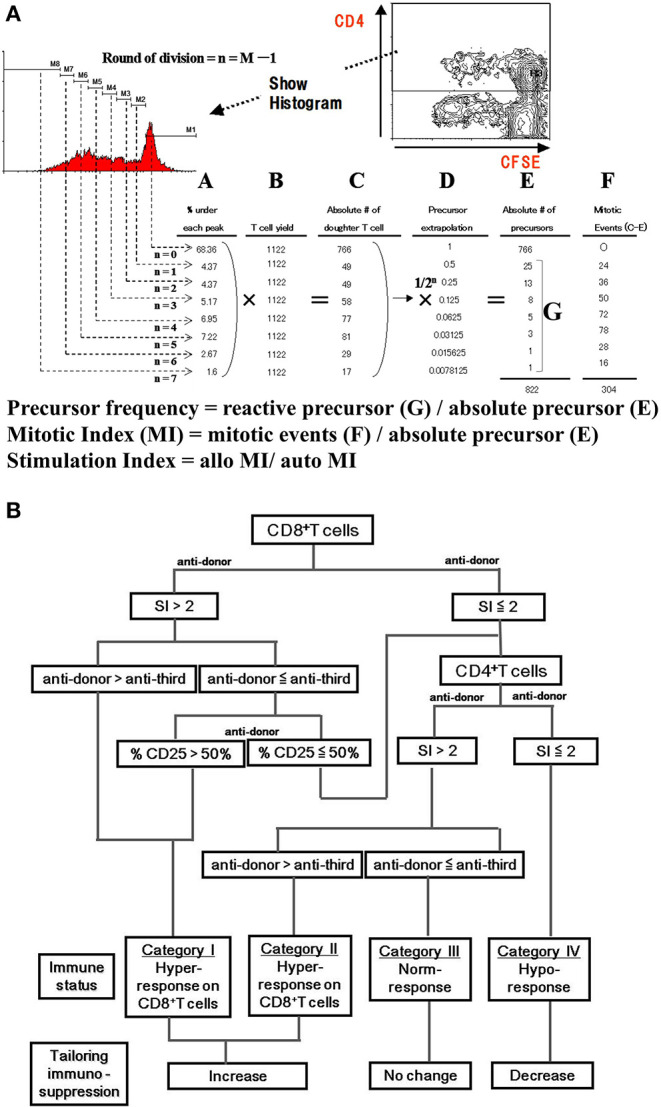
CFSE-MLR for immune monitoring in transplantation. **(A)** Intensity-based analysis of mixed lymphocyte reaction assay with CFSE dye (CFSE-MLR) provide quantitative estimation of the alloresponse. In brief, the plot and histogram show the gating strategy for CD4^+^ proliferating cells. Cell division are gated by the rationale that the CFSE fluorescence intensity shows the half-value from former generation. (A) Percentage of CD4+ T cell events in each division, (B) T cell yield, (C) the number of daughter T cells that had divided *n* times (A multiply B), (D) precursor extrapolation Using mathematical relationship, the number of division precursors (E,G) is extrapolated from the number of daughter cells of each division and from mitotic events (F). These values are used to calculate precursor frequency and mitotic index (MI). As normalized quantitative estimation, stimulation index are calculated by dividing MIs of allogeneic combinations by MIs of autologous controls. **(B)** Algorithm to estimate anti-donor alloreactivity in liver transplant recipients. The immune reactivity of liver transplantation recipients is classified into four categories. By analyzing the proliferation and CD25 expression of the CD4^+^ and CD8^+^ T-cell subsets in response to anti-donor and anti-third party stimuli, the immune status is categorized as hypo-, normo-, or hyper-responsive. In recipients with hyper-response on either CD4^+^ or CD8^+^ T cells, immunosuppressants consider to be increased. In patients with normo-response, immunosuppressant tapering is abandoned. Only in patients with hypo-response, immunosuppressant therapy can be tapered off (98). SI, stimulation index.

Along with the immune phenotype, functional assays have been investigated mainly using mixed lymphocyte reaction (MLR) assays with various readouts. One-way MLR with whole peripheral blood mononuclear cells (PBMCs) has been often attempted to use as clinical assay monitoring donor specific response. However, MLR readout with tritiated thymidine incorporation shows little predictive value because of its low level of reproducibility (93). ELISPOT and qPCR-based detection of cytokines in MLR assay showed sensitive results, but readout of limited cytokines from bulk cultured cells may be difficult to interpret as representative of the entire alloresponse (94–96). Non-toxic intracellular fluorescent dyes such as carboxyfluorescein diacetate succinimidyl ester (CFSE) stably stain intracellular proteins, and the fluorescence of each stained cell segregates equally to daughter cells upon cell division, resulting in sequential halving of cellular fluorescence intensity with each successive generation (97). This sequential halving of fluorescence can be analyzed to track cell division in populations of proliferating cells using intensity based analysis by flow cytometry (FACS) even in alloresponse which is comparatively lower incidence. Additionally, FACS analysis provides opportunity to assess detail phenotype of proliferating cell along with number of cells originally proliferated, that is halving of fluorescence is visualized as distinct peaks or populations of cells and can be used to track cell division in populations of proliferating cells. This allows phenotypic analysis of proliferating cells in addition to determining the number of cells produced in each generation by multicolor FACS analysis, that is, the precursor frequency of each CD4^+^ and CD8, the precursor frequency of each CD4^+^ and CD8^+^ T cell (and others) can be quantified separately (Figure 3A). The lack of proliferation in anti-donor MLR reflects the suppression of the anti-donor response (99). We have previously reported that optimization of immunosuppressive therapy based on the CFSE-MLR assay provides a low incidence of acute rejection, reduction of infectious complications, and helps in monitoring anti-self-response of CD4^+^ T cells, which predicts the recurrence of autoimmune liver diseases after LT (98, 100–102) (Figure 3B). In addition, CFSE-MLR-based immune monitoring has been proven to be a useful tool to personalize IS therapy, especially for LT patients with impaired renal function and HBV-infected LT patients requiring post-transplant HBV vaccination (103, 104). The benefit of CFSE-MLR immune-monitoring can be applied to T cell receptor (TCR) repertoire analysis by high-throughput sequencing. The Colombia group developed a TCR sequencing-based analysis of responding T cells in CFSE-MLR to identify and track a significant fraction of alloreactive T cell repertoire in any donor-recipient pair (105, 106). They have shown that liver-induced clonal deletion detected by tracking alloreactive TCR clones in pre-transplant MLR may contribute to achieving tolerance in LT recipients (107). Furthermore, another potentially beneficial application of MLR is the detection of activating induced markers and cytokines. CD154 (CD40L) has been reported to rapidly upregulate Ag-specific activating markers of T cells (108, 109). Upregulation of CD154 in T cells in MLR with donor stimulator was reported as a risk factor for rejection in pediatric liver transplant recipients (110). Like CD154, CD137 (4-1BB) has been reported as a specific activation-induced molecule on T cells (111). Interestingly, their combination, CD154^neg^CD137^+^, in CD4^+^ T cells have been reported to be representative of activated Tregs under Ag stimulation, including allo-stimulation, suggesting that it could be a candidate for monitoring alloreactive T cell responses in LT recipients (112, 113).

## Immune Tolerance Mediated by TREGS

### Applicability of Treg Cell Therapy in Liver Transplantation

Since the discovery of suppressive T cells and markers, Tregs (mostly defined as CD4^+^CD25^+^FOXP3^+^) have been shown to be key mediators in the induction and maintenance of immune tolerance through multiple mechanisms (114–116). Together with these accumulating findings in basic science and clinically reported footprints e.g., the number of Tregs are increasing in tolerant recipients, Treg-based cell therapy has been attempted for tolerance in the field of transplantation. Initial attempts have been made in the field of bone marrow transplantation and have shown the feasibility of transferring polyclonally expanded Tregs for graft vs. host disease (GVHD) prophylaxis (117–119). Together with promising rationale, several clinical trials have been conducted in LT recipients. Key considerations of this cell therapy are: (1) timing to infuse the cell product, (2) induction therapy to make space for adoptive Tregs, (3) cell component, whether Treg-enriched cell product or isolated Tregs for culturing, and (4) Ag specificity during expansion, polyclonal or donor specific (Table 2). In 2016, the Hokkaido University group demonstrated the impact of Treg-enriched cell therapy for inducing operational tolerance in 10 living donor LT patients (120). Autologous Treg-enriched cells were cultured in MLR in the presence of CD80/86 costimulatory blockade, and the cell product was administered after pre-conditioning with cyclophosphamide at early post-transplant period. Although three recipients with autoimmune liver disease developed cellular rejection during immunosuppressant weaning, the other seven (70%) recipients were successfully weaned off immunosuppressive drugs 18 months after liver transplantation. In spite of a small cohort, the result that all tolerant patients maintained normal graft function without immunosuppressive drugs for over 5 years is promising for Treg cell therapy for tolerance induction (121). Currently, clinical studies with isolated Tregs, rather than bulk cultured cells, are in operation. The King's college group is running a phase I/II clinical trial with a polyclonal expanded Treg isolated by a magnetic isolation system in LT patients with anti-thymocyte globulin (ATG) pre-conditioning (NCT02166177). No serious adverse events have been observed to date (122). The UCSF group conducted clinical trials using donor allo-Ag reactive Tregs (darTregs) cultured with donor-derived stimulators (NCT02188719). The protocol includes the use of ATG before the infusion of donor allo-Ag reactive Tregs (123). The Massachusetts General Hospital (MGH) group is employing costimulatory blockade-induced allospecific Tregs that are generated in short-term MLR with belatacept and isolated by magnetic isolation before administration. These three trials reduced the calcineurin inhibitor (CNI) regimen with the addition of an mTOR inhibitor before attempting complete IS withdrawal. Since clinical-grade manufactured Tregs cells are challenging, the King's college group treated 3 recipients finally out of the initial 23 and the USCF group's trial was terminated because of the manufacturing problem. USCF is conducting another trial, the ARTEMIS trial (NCT02474199), which has a different design, to aim at the reduction of CNI in patients with stable liver graft function in 2–6 years after LT with darTregs (123). It remains unclear when and what kind of Treg cell therapy is beneficial for LT recipients. Ongoing trials may clarify some points, but a systematic approach to investigate the best option may be needed.

**Table 2:** Trials of Treg cell therapy for liver transplantation.

**Institution**	**Trial ID**	**Type of trial**	***n***	**Timing of infusion after transplantation**	**Induction therapy**	**Cell isolation**	**Specificity**	**Type of Tregs**	**Current status**
Hokkaido	UMIN000015789	Phase I/II	10	2 weeks	Cyclophosphamide	No	Donor specific	Treg enriched donor-specific-anergic T cells	Published in 2016
UCSF	NCT02188719	Phase I	15	2–6 months	ATG	Yes	Donor specific	Donor alloantigen reactive Treg	Terminated
Beth Israel	NCT02739412	Phase II	7	2–4 years	–	–	–	Endogenous Treg by low dose IL-2 injection	Active, not recruiting
Nanjing	NCT 01624077	Phase I	1	–	–	No	Polyclonal	*In vitro* induced Treg	Unknown
UCSF	NCT02474199	Phase I/II	14	2–6 years	ATG	Yes	Donor specific	Donor-alloantigen reactive Treg	Completed
King's College	NCT02166177	Phase I/II	9	2 months	ATG	Yes	Polyclonal	Autologous Treg	Completed
MGH	NCT03577431	Phase I/II	9	2-6 months	Cyclophosphamide	Yes	Donor specific	Alloantigen-reactive Treg	Recruiting

*Treg, regulatory T cell; UCSF, University of California, San Francisco; ATG, anti-thymocyte globulin; MGH, Massachusetts General Hospital*.

### mTOR Inhibitor for Tolerance and Treg Expansion

Currently, a CNI-based regimen is widely employed as standard IS therapy for the management of liver transplantation. One of the most problematic side effects is nephrotoxicity of CNIs because LT candidates frequently have a variety of degrees of renal dysfunction, and chronic renal failure has a negative impact on long-term outcomes. The strategy of early CNI minimization and mTOR inhibitor maintenance has been attempted to achieve better renal function after liver transplantation. Meta-analysis and recent RCTs have shown a protective effect on renal function by converting CNIs into mTOR inhibitor, but also high frequency of rejection compared to conventional CNI-based therapy, suggesting that selected patients could receive the benefit of mTOR inhibitor conversion (124–126). The mTOR signaling pathway through PI3K/AKT is widely utilized in the regulation of cellular activity in immune cells and cancer cells. mTOR inhibitors have been reported to have therapeutic effects on hepatocellular carcinoma (HCC) through multiple mechanisms, including direct antitumor effects and immune regulation (127–129). According to the antitumor effect, LT recipients with HCC may be good candidates for mTOR inhibitor regimen (130). Another topic in the transplantation field of mTOR signaling is the impact on Treg stability and function, usually mTOR inhibition recognized as favorable effects (131). One recent IS withdrawal trial has been conducted expecting this “Treg friendly effect” to induce operational tolerance (74). Further investigation is required to elucidate the clinical application of mTOR inhibitors for transplantation tolerance.

## Outlook on Employing SNPs and miRNAs for Tolerance

Genetic factors have been reported to be involved in the mechanisms of transplant tolerance and rejection (132). Here, we summarize recent advances in genetics and genomics, particularly single nucleotide polymorphisms (SNPs) and microRNAs (miRNAs), and their roles intolerance after LT.

### Genome-Wide Association Studies (GWAS)

Recent GWAS have established the genes and variants associated with outcomes in transplantation patients. Multiple GWAS have been conducted since 2016 on solid-organ transplantation, including acute rejection in renal transplantation, post-transplant malignancy in heart or renal transplantation, long-term allograft function, and new-onset diabetes mellitus after renal transplantation; however, there are no GWASs on liver transplantation (96, 132).

### Candidate Gene Approaches (CGA)

The candidate gene approach has been applied in liver transplantation by conducting genetic association studies focusing on associations between immune-associated genetic variation and graft survival/rejection incidence. HLA-G, a non-classical HLA-class, has been associated with increased graft survival and decreased number of rejection cases (133–135). It is also known that HLA-G is capable of inducing a new generation of regulatory Tregs (136). A recent study has demonstrated that 14-bp ins/ins and +3142GG genotypes of HLA-G, which seem to be of serious importance for HLA-G expression, in LT recipients are involved in a low risk of acute rejection in liver transplantation, suggesting that LT recipients with a lower for developing an acute rejection may be identified by application of these genotypes as biomarkers (137). Another report has shown that the donor liver tissue-derived CYP3A5 rs776746 and small ubiquitin-like modifier 4 (SUMO4) rs237025 SNPs are associated with tacrolimus pharmacokinetics in the early period after LT, suggesting that combined evaluation of these donor genotypes may help determine the withdrawal or elimination of tacrolimus (138). We have also reported that the FOXP3 gene rs3761548 A/C SNP in living donor LT recipients is significantly concerned with susceptibility to steroid-resistant acute rejection and dnDSA formation, suggesting that the IS regime and/or anti-rejection treatment regimen should be adjusted on an individual basis by identifying FOXP3 SNPs (139). These genetic association studies may hopefully provide immune-related SNPs that can be useful markers to reduce or withdraw immunosuppressive drugs.

### miRNAs as Biomarkers

miRNAs, which are ~20–22 nucleotide single-stranded RNA species, and play a central role in the regulation of protein-coding genes, are also emerging as robust biomarkers for assessing allograft status. Millán et al. have reported that plasma miRNAs can serve as early non-invasive prognostic and diagnostic biomarkers for T-cell mediated acute rejection in LT recipients, that is, miR-155-5p regulates the differentiation of CD4^+^ T cells into Th cells and IFN-γ production in human T and NK cells, and miR-181a levels modulate T cell receptor sensitivity and intensity of signaling (140). Hence, the plasma levels of miR-155-5p and miR-181a-5p after LT potentially help identify patients for IS minimization. Revilla-Nuin et al. have reported a set of differentially expressed miRNAs in tolerant recipients after liver transplantation that might promote and control the activation of Tregs necessary to develop operational tolerance (141). Their study showed that miR95, miR24, miR31, miR146a, and miR155 were expressed more in tolerant than in non-tolerant recipients, and were positively correlated with activated Treg markers. These five miRNAs were upregulated in the peripheral blood of LT recipients, and the transcription factor Foxp3 was associated with the miRNA profiles. miR155 is constitutively expressed in Tregs; Foxp3 binds to the promoter of miR155 in the B cell integration cluster and maintains the elevated levels of miR155 required for Treg proliferation. Furthermore, Vitalone et al. reported increased expression of miR-142-5p and miR-181a in tolerant livers in an allogeneic rat LT model (142). Morita et al. have also identified miRNAs involved in acute rejection and spontaneous tolerance in murine hepatic allografts (143). They found that miR-146a, 15b, 223, 23a, 27a, 34a, and 451 were upregulated in the allogenic liver grafts compared with the expression observed in the syngeneic grafts, whereas miR-101a, 101b, and 148a were downregulated, demonstrating the change of miRNAs in the allografts and may suggest the role of miRNAs in the induction of tolerance after liver transplantation.

## Conclusion

Progresses in immunosuppressive therapy have efficiently reduced the incidence of acute rejection of liver allograft. However, life-long IS has inevitably led to substantial morbidity and mortality. Thus far, trial and error have been attempted to minimize or even withdraw immunosuppressants in select patients. These attempts would be more successful through the establishment of reliable immune-monitoring methods and biomarkers. In addition, deliberate immunomodulatory interventions would further improve the outcome of these attempts. This review has summarized our knowledge of mechanisms underlying immune-tolerance induced after liver transplantation and prospective strategies to intentionally complete withdrawal of IS treatment.

## Author Contributions

NT participated in the role of drafting and revising. MO, HT, KI, YT, and TO participated in roles of writing original draft. HO participated in roles of concepts, design, and drafting and revising. All authors contributed to the article and approved the submitted version.

## Conflict of Interest

The authors declare that the research was conducted in the absence of any commercial or financial relationships that could be construed as a potential conflict of interest.
